# Robotic development: ‘patients’ safety always comes first’

**DOI:** 10.1093/icvts/ivac057

**Published:** 2022-03-31

**Authors:** Matthieu Sarsam, Gerardo Mordojovich, Benjamin Bottet, Jean-Marc Baste

**Affiliations:** Department of General and Thoracic Surgery, Rouen Teaching Hospital, Rouen, France

**Keywords:** Robotic surgery, Patient safety, Innovation

We thank the editor for his kind invitation to write a commentary regarding ‘Emergency rollout and conversion procedures during the three-arm robotic open-thoracotomy-view approach’ by Sakakura *et al*. [1].

In this article, the authors describe a novel robot-assisted thoracic approach based on their experience with vertical thoracotomies, and the principles of their current video-assisted thoracic surgery (VATS) technique. The article also presents a thoroughly written emergency roll-out procedure in the form of a checklist in case of major or minor incidents, and possible conversion ways to VATS or thoracotomy (vertical or lateral).

Minimally invasive techniques started getting more popular since the first anatomic lung lobectomy was performed by Roviaro in Italy in October 1991. Many authors report less pain and complications in comparison to open approaches with similar or non-inferior oncological results [2].

Since then, more dedicated instruments and scopes were and continue to be developed in this domain, allowing for more complex procedures to be performed reaching the level of carinal resections by uniportal approaches in expert hands [3].

A new era in thoracic surgery has begun with the surgical robotic technology; with the high-quality three-dimensional zoomed view allowing micro-dissection, the ability to use indocyanine green and the 7° of movement of the EndoWrist instruments, some centres nowadays report going from open to robot-assisted approach even without passing by VATS.

However, with these newer and less-invasive approaches, the issue of patients’ safety becomes more fundamental than ever, especially in the light of major or catastrophic intraoperative complications [4, 5], and the question of an emergency protocol, that is well written with defined roles for each member, and conversion roll-out is of utmost importance. This is especially true in Robot-assisted thoracic surgery (RATS) where managing vascular injuries during major resections could be more stressful for the senior surgeon sitting on the console, away from the patient.

In our point of view, safety has 2 broad definitions: the first is the oncological quality of the major lung resection and the second is the acute haemodynamic shock in case of a major vascular injury and bleeding.

Since the early beginnings of our minimally invasive programme back in 2008, we started developing an internal debriefing system after each incident. At this stage, our focus was on the root causes analysis and managing the technical aspects of these issues [6], thoroughly reviewing patients’ imagery for missed anatomic variation and even changing dissection techniques if required. A video recorder was integrated into our operative room, and systematic recording of all lung anatomic resections was implemented; this serves as an operative ‘black box’.

Impacted by major publications [7, 8], a nationwide obligatory checklist practice was enforced in France in 2010. Aside from obvious elements (names, gender, operation to be practiced), our team’s revised version includes the definition of the risk of conversion, the name of the (friend) to call in case of troubles, any anatomical variation detected on the preoperative CT scan or the 3D reconstructions.

With time and experience, our Simulation Program was developed in 2014. During these sessions, a full-scale multidisciplinary management of a major preoperative Haemorrhage is practiced, in a high-fidelity model with all team debriefing afterwards. An external expert in aviation safety audits the process.

In 2016, Cao *et al.* [9] published the principles of Poise, Pressure, Prepare, and Proximal control, while we believe that technical skills are highly important; we would like to emphasize the importance of non-technical skills (team communications, situational awareness and personal resources management) in the successful management of such crisis [7].

Our central message is that team-based simulation remains the cornerstone in such conditions leading to the management of more complex situations as dangerous as preoperative cardiac arrests [10].

We organize 2 simulation sessions yearly as we take into account the turnover of residents, surgical assistants and the necessity to keep the team up to date with the latest publications and insights regarding the issue [11].

We congratulate the authors for the quality of their emergency rollout checklist; however, we do believe that having >1 conversion thoracotomies could be confusing. Cool injuries can sometimes transform into catastrophic ones very rapidly. The emergency thoracotomy seems to be in the 7th intercostal space rendering vascular control of upper lobe vessels rather challenging. The team reports rehearsing their protocols in a frequent manner; there is unfortunately no description of the procedure, the periodicity and the precise objectives.

More simulation sessions on cadavers or animals would be of high importance to validate the emergency rollout protocol in this case.

We also encourage the authors to keep the thoracic surgery community informed with more complex cases and experience with future conversions to thoracotomy.

## References

[1] Sakakura N , Nakada T, Shirai S, Takahara H, Suzuki A, Takahashi Y et al Emergency rollout and conversion procedures during the three-arm robotic open-thoracotomy-view approach. Interact Cardiovasc Thorac Surg 2022;34:1045–51. 10.1093/icvts/ivab336 PMC9159417 34849975

[2] Flores RM , Park BJ, Dycoco J, Aronova A, Hirth Y, Rizk NP et al Lobectomy by video-assisted thoracic surgery (VATS) versus thoracotomy for lung cancer. J Thorac Cardiovasc Surg 2009;138:11–8. 19577048 10.1016/j.jtcvs.2009.03.030

[3] Gonzalez-Rivas D , Fieira E, Delgado M, de la Torre M, Mendez L, Fernandez R. Uniportal video-assisted thoracoscopic sleeve lobectomy and other complex resections. J Thorac Dis 2014;6:S674–81. 25379210 10.3978/j.issn.2072-1439.2014.09.17 PMC4221345

[4] Decaluwe H , Petersen RH, Hansen H, Piwkowski C, Augustin F, Brunelli A et al; ESTS Minimally Invasive Thoracic Surgery Interest Group (MITIG). Major intraoperative complications during video-assisted thoracoscopic anatomical lung resections: an intention-to-treat analysis. Eur J Cardiothorac Surg 2015;48:588–98, discussion 599. 26385060 10.1093/ejcts/ezv287

[5] Video-assisted thoracoscopic surgery (VATS) lobectomy: catastrophic intraoperative complications. https://pubmed.ncbi.nlm.nih.gov/22014713/. 10.1016/j.jtcvs.2011.09.028 22014713

[6] Johna S , Tang T, Saidy M. Patient safety in surgical residency: root cause analysis and the surgical morbidity and mortality conference—case series from clinical practice. Perm J 2012;16:67–9. 10.7812/tpp/11-097 PMC3327116 22529763

[7] Arriaga AF , Bader AM, Wong JM, Lipsitz SR, Berry WR, Ziewacz JE et al Simulation-based trial of surgical-crisis checklists. N Engl J Med 2013;368:246–53. 23323901 10.1056/NEJMsa1204720

[8] A surgical safety checklist to reduce morbidity and mortality in a global population. https://pubmed.ncbi.nlm.nih.gov/19144931/. 10.1056/NEJMsa0810119 19144931

[9] Cao C , Cerfolio RJ, Louie BE, Melfi F, Veronesi G, Razzak R et al Incidence, management, and outcomes of intraoperative catastrophes during robotic pulmonary resection. Ann Thorac Surg 2019;108:1498–504. 31255610 10.1016/j.athoracsur.2019.05.020 PMC6889954

[10] Crisis checklist (Code Red) for the management of cardiac arrest during minimally invasive thoracic surgery: case report. https://pubmed.ncbi.nlm.nih.gov/32677971/. 10.1186/s13019-020-01200-4 PMC7367320 32677971

[11] Baste J-M , Bottet B, Selim J, Sarsam M, Lefevre-Scelles A, Dusseaux M-M et al Implementation of simulation-based crisis training in robotic thoracic surgery: how to improve safety and performance? J Thorac Dis 2021;13:S26–34. 34447589 10.21037/jtd-2020-epts-03 PMC8371544

